# Major salivary gland aplasia and hypoplasia in Down syndrome: review of the literature and report of a case

**DOI:** 10.1002/ccr3.975

**Published:** 2017-05-04

**Authors:** Mary Jane Chadi, Guy Saint Georges, Francine Albert, Gisele Mainville, Julie Mi Nguyen, Adel Kauzman

**Affiliations:** ^1^ Faculty of DentistryUniversité de MontréalMontréalQuébecCanada; ^2^ Private practiceLavalQuébecCanada

**Keywords:** Congenital abnormalities, Down syndrome, parotid gland, salivary glands, tooth wear, xerostomia

## Abstract

Salivary gland aplasia and hypoplasia are rarely described in the medical literature. This article presents a case of aplasia and hypoplasia of the major salivary glands in a patient with Down syndrome. A literature review, as well as an overview of the diagnosis and management of this condition, is presented.

## Introduction

Aplasia and hypoplasia of the major salivary glands are rare developmental anomalies. They can be isolated or part of a syndrome, as well as unilateral or bilateral. They can affect more than one of the major glands 1, 2.

Salivary glands originate from the first branchial arches. The parotid and the submandibular glands begin their development at approximately 4–6 weeks of intrauterine life, while the sublingual and minor glands form during the 8th to 12th weeks of gestation 3. Proliferation and down‐growth of the oral epithelium into the underlying mesenchyme will form a network of epithelial cords through *branching morphogenesis*. Lumina will subsequently form within these cords creating the excretory pathways of the salivary glands. The inner epithelium at the distal end of the branching cords will differentiate to form secretory cells. These could be serous or mucous depending on the developing gland. The peripheral epithelium at the distal end of these cords will form the myoepithelial cells. Contraction of these cells directs saliva out of the acini into the ducts 3, 4.

The parotid glands produce almost exclusively serous saliva and are most active during salivary flow stimulation. Parotid secretion accounts for more than 50% of stimulated saliva and only 28% of unstimulated saliva 4. The submandibular gland produces mixed saliva with both mucous and serous components. The sublingual gland represents the smallest of the major glands and it produces purely mucous saliva. Together, the submandibular and sublingual glands produce 68% of the unstimulated saliva. Minor salivary glands produce mucous saliva in small proportions 5.

Saliva is crucial for maintaining oral homeostasis. Its antimicrobial properties inhibit the establishment of a pathologic flora while the buffering effect of bicarbonate neutralizes the acid produced by cariogenic bacteria. Saliva plays a role in the remineralization of teeth by bringing calcium, phosphates, fluoride, and peptides to the enamel surface. Its role in maintaining proper oral hygiene is crucial as it allows for an auto‐cleansing effect and easier hygiene procedures. Lubrication provided by serous saliva is essential for tasting, swallowing, and to maintain oral comfort. Saliva plays a role in digestion through the production of salivary amylase and lipase 3.

Down syndrome (DS) is a genetic disorder associated with increasing maternal age with an overall incidence of 1/800 live births 6. Characteristic oral and maxillofacial features of patients affected by DS are presented in Table 1. The underlying systemic disorders that affect these patients can lead to a number of oral manifestations. For example, undiagnosed celiac disease can lead to enamel defects and aphthous stomatitis 7, underlying leukemia can lead to hemorrhagic gingival hyperplasia and ulcerations 8, 9, and diabetes can cause severe periodontitis 10. Early detection and management of these manifestations can improve quality of life and prognosis of affected patients.

**Table 1:** Common maxillofacial signs of Down syndrome 8

Facial	Upslanting palpebral fissures, brachycephaly, epicanthal folds, flattened nose bridge, flattened facies, muscle hypotonia, protruding tongue, mouth breathing
Dental	Hypodontia, hyperdontia, microdontia, taurodontism, crown variants, delayed eruption
Periodontal	Gingivitis, premature periodontitis and exfoliation of the teeth
Mucosal	Cheilitis, macroglossia, erythema and signs of infection
Skeletal	Clefts, malocclusion, TMJ dysfunction

A recent study by Chaushu et al. suggests that salivary flow is decreased in patients with DS even if there is a clinical impression of sialorrhea 11. Despite the fact that xerogenic medications, age, and institutionalization had a negative impact on flow rate, salivary output reduction was well recognized as being an integral part of the syndrome 11. In patients with DS, only a few cases of hyposalivation caused by salivary gland underdevelopment have been reported in the English medical literature. We present a rare case of bilateral major salivary gland aplasia and hypoplasia in a patient with DS presenting with severe oral dryness.

## Case Study

**Figure 1:**
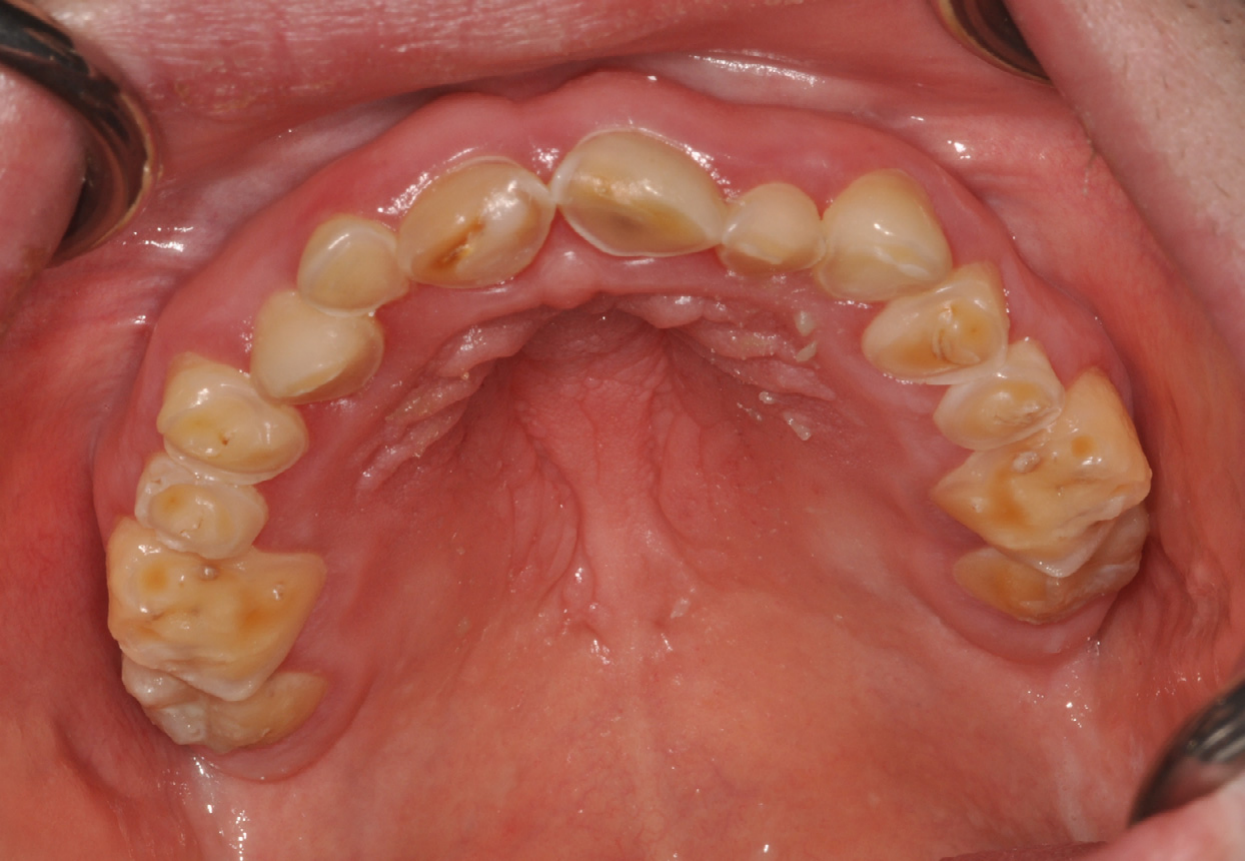
Severe erosion of the maxillary dentition.

**Figure 2:**
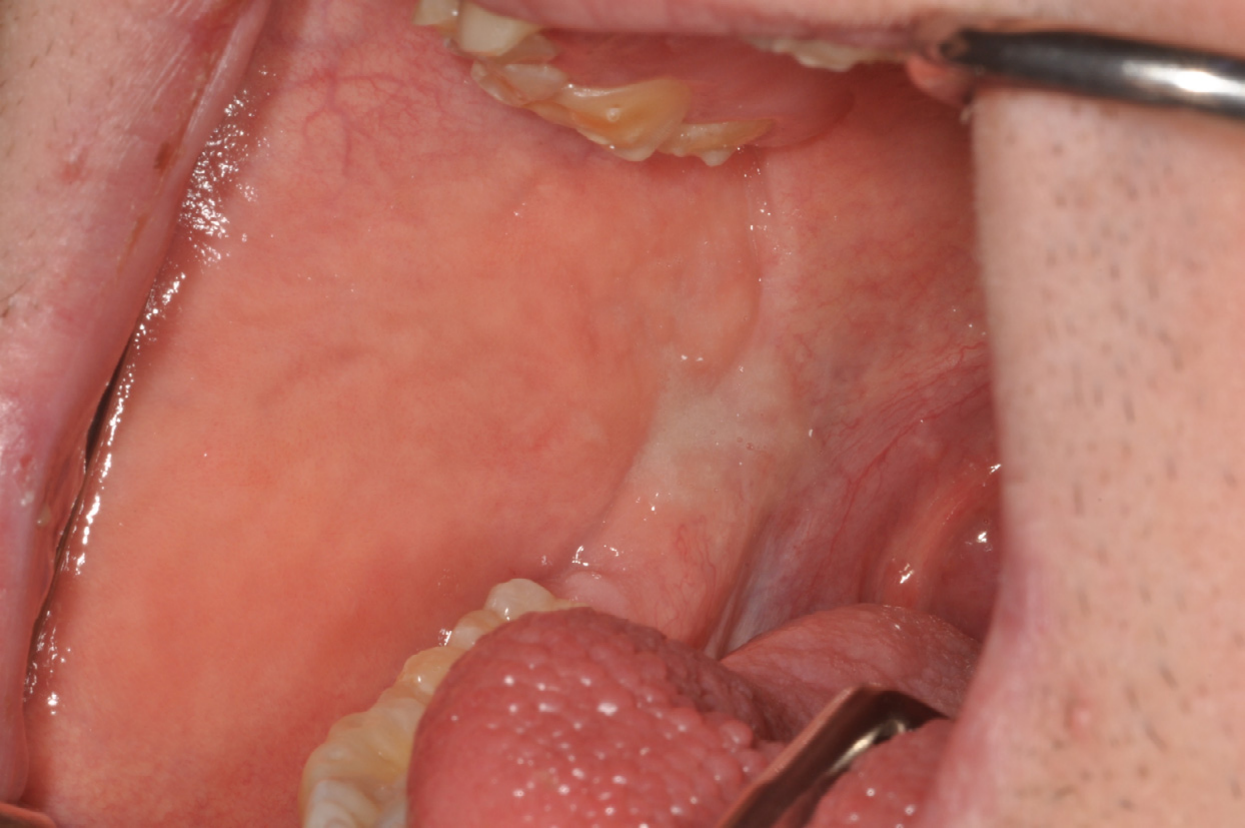
Absence of Stensen's duct orifice on the right buccal mucosa. A similar appearance was noted on the left buccal mucosa.

A 23‐year‐old patient with DS was referred for prosthodontic rehabilitation and for the correction of poor aesthetics and severe loss of tooth substance affecting the maxillary dentition. Review of systems revealed a history of celiac disease, colitis, and hypothyroidism. Clinical examination showed objective mucosal dryness, a large fissured tongue, and severe erosion and attrition of the maxillary dentition **(**Fig. 1). There was generalized erythema and edema of the gingival tissues and oral mucosae. Stensen's duct openings could not be located on buccal mucosae **(**Fig. 2), and no saliva could be expressed upon palpation of the parotid and submandibular gland regions. A diagnosis of chronic erythematous candidiasis was established and a congenital absence of the parotid glands was strongly suspected. Management included topical antifungal treatment, biannual regular dental examinations, proper oral hydration, use of an occlusal splint, and fluoride gel application in custom trays 5 min before bedtime. Complete dental rehabilitation was not recommended essentially due to the poor long‐term prognosis of the teeth.

**Figure 3:**
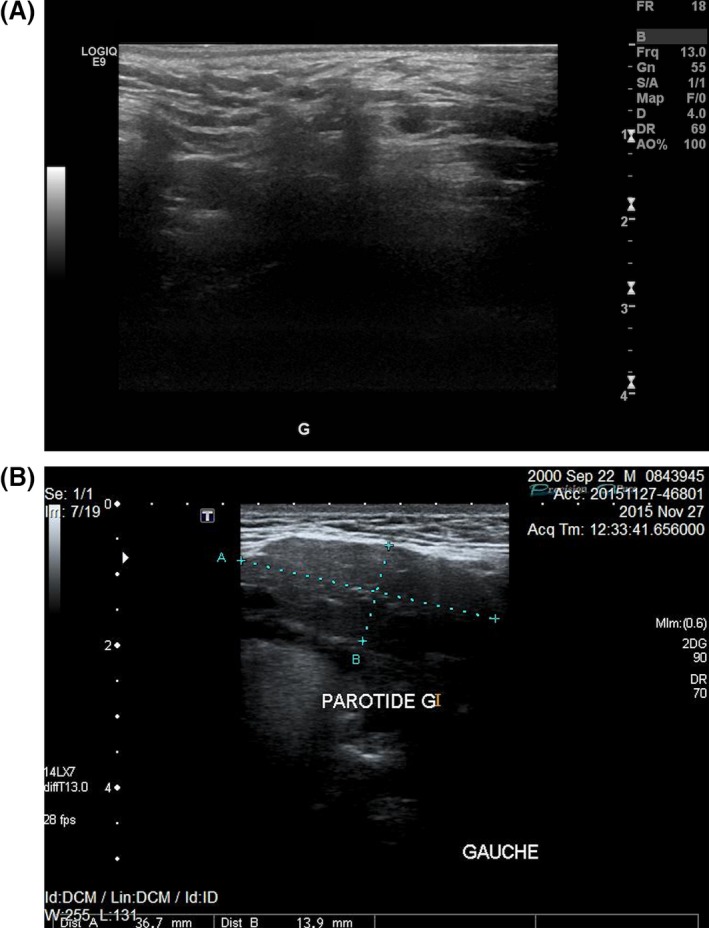
Ultrasonography demonstrating the complete absence of the left parotid gland in the presented case (A) compared to the ultrasonography of a normal parotid gland in a healthy 16‐year‐old male (B).

**Figure 4:**
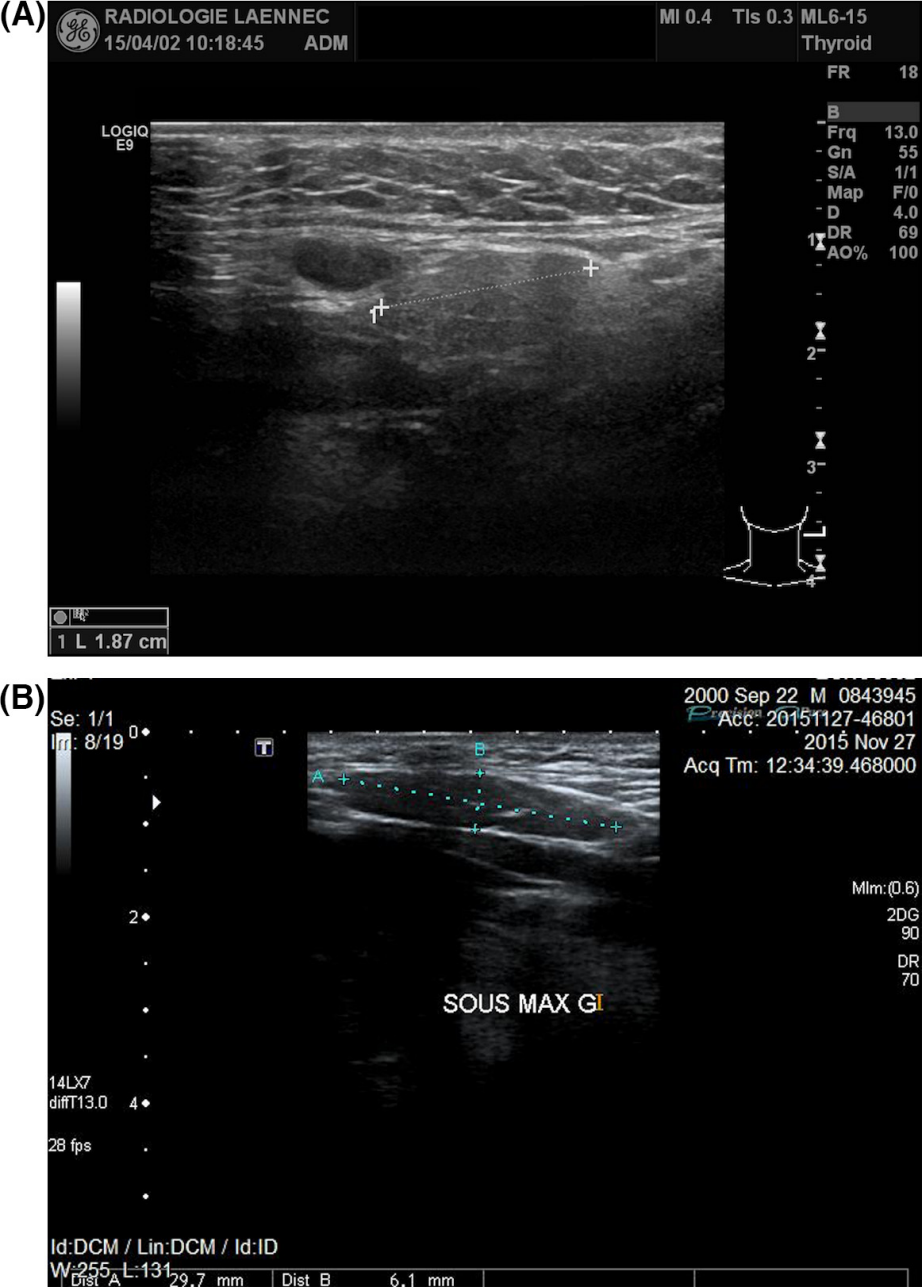
Ultrasonography demonstrating the hypoplastic left submandibular gland in the presented case (A) compared to the ultrasonography of the left submandibular gland in a healthy 16‐year‐old male (B).

Salivary gland ultrasonography was performed, and upon cervicocephalic examination, both parotid glands were undetected (Fig. 3A and B). The right submandibular gland measured 24 mm and the left measured less than 18 mm. The submandibular glands were described as hypoplastic considering that under normal circumstances, they measure on average 30 mm anterior‐posteriorly (Fig. 4A and B) 12. A diagnosis of salivary gland aplasia and hypoplasia was rendered.

## Discussion

Very few cases of salivary gland aplasia and hypoplasia have been reported in the literature. A review by Matsuda et al. 13 states that the first case report of unilateral submandibular aplasia was described by Grüber in 1885 following a cadaver dissection. From 1885 to 1999, Matsuda et al.'s review identified 44 cases of major salivary gland aplasia of which 34 had bilateral aplasia. A familial pattern was observed in six patients from three different families.

A similar review carried out in 2010 by Chaushu et al. 11 reported a total of 19 new cases from 1999 to 2010. Among those, the authors compiled six cases of bilateral aplasia of the parotid gland, six cases of bilateral aplasia of the parotid and submandibular glands, and four cases of bilateral aplasia of the submandibular gland. The remaining three cases represented unilateral aplasia or significant reduction in the salivary flow rate. Six of the 19 cases reported were associated with other developmental anomalies such as lachrymal and ectodermal defects while one case was associated with DS.

In 2010, N. Pham Dang et al.'s review article reported 34 cases of salivary gland aplasia since Grüber's first report in 1885 2. Some of the cases reported were mentioned in Matsuda's review while others had been published after 1999. Of the 34 cases, nine had bilateral submandibular gland aplasia and only one reported bilateral agenesis of the parotid. Thirteen patients in this review presented congenital absence of unspecified major salivary glands and had anomalies of the lacrimal puncta, suggesting an association with Lacrimo‐auriculo‐dento‐digital (LADD) syndrome.

It is important to note that the three review articles did not mention the inclusion and exclusion criteria nor the key words used to perform the literature search. In addition, publications in which aplasia and hypoplasia of the major salivary glands are a secondary focus were not included.

Salivary gland aplasia and hypoplasia is not a recognized manifestation of DS. For this reason, a limited number of cases are reported in the English‐language literature. In 2004, Ferguson et al. published a case of bilateral parotid aplasia in a 24‐year‐old patient with DS 14. The patient in this study had an overall good health but presented important signs of hyposalivation. A bilateral depression was observed in the periauricular area and no Stensen's duct orifices were located in the buccal mucosae. A ^99m^Tc‐pertechnetate scan showed no uptake in the parotid area as opposed to the submandibular glands or thyroid regions.

A cross‐sectional study published in 2013 by M. Odeh compared the presence or the absence of major salivary glands in 31 healthy children and 15 patients with DS 15. Using ultrasonography, it was reported that only four patients with DS had aplasia of one or more salivary gland compared to none in the control group. Three other patients with DS showed hypoplasia of the parotids and/or the submandibular glands, but one of those three patients had severe facial burns and scarring, which could have affected the size of his parotid gland. These results suggest that there might be an association between salivary gland aplasia/hypoplasia and DS. However, additional studies involving a larger number of patients have to be conducted in order to further investigate this possibility.

The initial diagnosis and the risk–benefit ratio are factors that determine the type of medical imaging ordered in a given clinical situation. Ultrasonography is an operator‐dependent method, which is widely used as a primary step to detect calculi, cysts, tumors, and dimensional changes of the salivary glands 16, 17. It was selected in this case because of its low cost, noninvasive nature, absence of radiation, adequate sensitivity, and simplicity in assessing the absence of the glands (Figs 3, 4A and B), especially in the presence of a high clinical suspicion index 18. Magnetic resonance imaging (MRI) and computed tomography (CT) scan can be used to detect such anomalies. However, the cost of these imaging techniques is high. MRI has the advantage of not exposing the patient to ionizing radiation. It is useful to characterize the tissue content and to obtain detailed information on a lesion. However, it is a very expensive imaging modality and the procedure itself can be troublesome to certain patients 16. CT scans expose patients to high doses of radiation. However, they represent an essential imaging modality and provide detailed information often needed to diagnose and treat invasive and noninvasive lesions 19. Sialography is prescribed to highlight the ductal system of a gland and to investigate ductal anomalies such as a blockage 16.

Hyposalivation is defined as the clinical state of salivary output reduction. This diagnosis is based on sialometric test results. Dry mouth or xerostomia is a symptom. These two concepts are closely related, yet they are rarely simultaneously recorded 20, 21. Before treating a patient with dry mouth symptoms, it is essential to investigate the underlying etiology and residual salivary gland activity. Management should be modulated depending on these two factors.

One of the most important causes of xerostomia is the long‐term use of xerogenic drugs. A large number of drugs, especially when combined, has been associated with reduced salivary production 22. Xerostomia is also a major complication of radiation therapy to the head and neck region 23. Autoimmune diseases such as Sjögren's syndrome (SS) can cause oral dryness. Other conditions such as diabetes, sarcoidosis, chronic graft‐versus‐host disease, hepatitis, HIV, and chronic anxiety have been reported to cause xerostomia 24. As mentioned earlier, salivary gland hypoplasia and aplasia are obvious causes of oral dryness; however, they are rarely seen in clinical practice.

Napeñas et al. suggest a stepwise and multidisciplinary approach to the treatment of xerostomia. Nonmedicinal topical agents and lubricants are easily accessible tools 17. The use of a humidifier, increasing water intake, and limiting diuretics or irritating foods should be suggested 17. If xerostomia is caused or aggravated by certain medications, the patient should consider another treatment alternative with his physician. However, if the outcome of these methods is not satisfying, a patient–doctor discussion regarding expectations toward treatment should follow, especially for patients with irreversibly damaged salivary glands 25.

Regular dental examinations should be scheduled at 4‐ to 6‐month intervals, and optimal oral hygiene should be integrated in the patient's daily routine 17, 21. Low sugar diets in conjunction with regular topical use of fluoride‐based dental products are important approaches to limit the incidence of decay. Custom trays can be fabricated for daily fluoride application 21. Pit and fissure sealants can be used in selected cases. Candidiasis, a common complication of xerostomia, can be treated using topical or systemic antifungals depending on the clinical context 17.

Parasympathomimetic drugs such as pilocarpine and cevimeline are generally reserved for patients with SS or those treated with radiation to the head and neck. These therapies have to be used cautiously because serious side effects, interactions with other medications, and aggravation of existing medical conditions such as gastric ulcers, uncontrolled asthma, and hypertension have been documented 17, 21.

## Conclusion

To our knowledge, this is the fourth case report assessing multiple gland hypoplasia and aplasia in patients with DS. This article highlights the importance of a dental practitioner's involvement in identifying and treating the maxillofacial manifestations of a systemic disease or a common syndrome. Further research with rigorous methods should be conducted in order to accurately determine the prevalence of major salivary gland aplasia and hypoplasia in the general population and in patients with Down syndrome.

## Patient Permission/Consent

Written permission was obtained from the patient and from the patient's mother.

## Authorship

MJC: wrote the first draft, edited all versions of the manuscript, and performed the literature review and data acquisition. AK: provided care for the patient, supplied clinical images, and reviewed and prepared all versions of the manuscript for submission. GSG: performed ultrasonography, provided all radiographic references, and critically reviewed the final manuscript. FA: was the initial caregiver of the patient and critically reviewed the final manuscript. GM and JMN: critically reviewed the final manuscript.

## Conflict of Interest

None declared.

## References

[1] Taji, S. S. , N. Savage , T. Holcombe , F. Khan , and W. K. Seow . 2011 Congenital aplasia of the major salivary glands: literature review and case report. Pediatr. Dent. 33:113–118. 21703060

[2] Pham Dang, N. , M. Picard , J. M. Mondie , and I. Barthelemy . 2010 Complete congenital agenesis of all major salivary glands: a case report and review of the literature. Oral Surg. Oral Med. Oral Pathol. Oral Radiol. Endod. 110:e23–e27. 20656534 10.1016/j.tripleo.2010.04.008

[3] Nanci, A. , and A. R. Ten Cate . 2013 Ten Cate's oral histology: development, structure, and function, 8th ed. Elsevier, St. Louis, MO. xiii, 379 pp.

[4] Tucker, A. S. 2007 Salivary gland development. Semin. Cell Dev. Biol. 18:237–244. 17336109 10.1016/j.semcdb.2007.01.006

[5] Proctor, G. B. 2016 The physiology of salivary secretion. Periodontol 2000 70:11–25. 26662479 10.1111/prd.12116

[6] Alldred, S. K. , J. J. Deeks , B. Guo , J. P. Neilson , and Z. Alfirevic . 2012 Second trimester serum tests for Down's Syndrome screening. Cochrane Database Syst. Rev. 6:1–215. 10.1002/14651858.CD009925 PMC7086392 22696388

[7] Pastore, L. , A. Carroccio , D. Compilato , V. Panzarella , R. Serpico , and L. Lo Muzio . 2008 Oral manifestations of celiac disease. J. Clin. Gastroenterol. 42:224–232. 18223505 10.1097/MCG.0b013e318074dd98

[8] Desai, S. S. 1997 Down syndrome: a review of the literature. Oral Surg. Oral Med. Oral Pathol. Oral Radiol. Endod. 84:279–285. 9377191 10.1016/s1079-2104(97)90343-7

[9] Hou, G. L. , J. S. Huang , and C. C. Tsai . 1997 Analysis of oral manifestations of leukemia: a retrospective study. Oral Dis. 3:31–38. 9456644 10.1111/j.1601-0825.1997.tb00006.x

[10] Khader, Y. S. , A. S. Dauod , S. S. El‐Qaderi , A. Alkafajei , and W. Q. Batayha . 2006 Periodontal status of diabetics compared with nondiabetics: a meta‐analysis. J. Diabetes Complications 20:59–68. 16389170 10.1016/j.jdiacomp.2005.05.006

[11] Chaushu, S. , A. Becker , G. Chaushu , and J. Shapira . 2002 Stimulated parotid salivary flow rate in patients with Down syndrome. Spec. Care Dentist. 22:41–44. 12014860 10.1111/j.1754-4505.2002.tb01208.x

[12] Dost, P. , and S. Kaiser . 1997 Ultrasonographic biometry in salivary glands. Ultrasound Med. Biol. 23:1299–1303. 9428127 10.1016/s0301-5629(97)00152-x

[13] Matsuda, C. , Y. Matsui , K. Ohno , and K. Michi . 1999 Salivary gland aplasia with cleft lip and palate: a case report and review of the literature. Oral Surg. Oral Med. Oral Pathol. Oral Radiol. Endod. 87:594–599. 10348519 10.1016/s1079-2104(99)70140-x

[14] Ferguson, M. M. , and Y. Ponnambalam . 2005 Aplasia of the parotid gland in Down syndrome. Br. J. Oral Maxillofac. Surg. 43:113–117. 15749210 10.1016/j.bjoms.2004.01.001

[15] Odeh, M. , M. Hershkovits , J. Bornstein , N. Loberant , M. Blumenthal , and E. Ophir . 2013 Congenital absence of salivary glands in Down syndrome. Arch. Dis. Child. 98:781–783. 23908188 10.1136/archdischild-2013-303841

[16] Yousem, D. M. , M. A. Kraut , and A. A. Chalian . 2000 Major salivary gland imaging. Radiology 216:19–29. 10887223 10.1148/radiology.216.1.r00jl4519

[17] Napenas, J. J. , M. T. Brennan , and P. C. Fox . 2009 Diagnosis and treatment of xerostomia (dry mouth). Odontology 97:76–83. 19639449 10.1007/s10266-008-0099-7

[18] Jousse‐Joulin, S. , V. Milic , M. V. Jonsson , A. Plagou , E. Theander , N. Luciano , et al. 2015 Is salivary gland ultrasonography a useful tool in Sjogren's syndrome? A systematic review. Rheumatology (Oxford) 55:789–800. 26667216 10.1093/rheumatology/kev385

[19] Drage, N. A. , J. E. Brown , M. P. Escudier , and M. McGurk . 2000 Interventional radiology in the removal of salivary calculi. Radiology 214:139–142. 10644113 10.1148/radiology.214.1.r00ja02139

[20] Villa, A. , A. Wolff , D. Aframian , A. Vissink , J. Ekstrom , G. Proctor , et al. 2015 World Workshop on Oral Medicine VI: a systematic review of medication‐induced salivary gland dysfunction: prevalence, diagnosis, and treatment. Clin. Oral. Investig. 19:1563–1580. 10.1007/s00784-015-1488-2 25994331

[21] Glore, R. J. , K. Spiteri‐Staines , and V. Paleri . 2009 A patient with dry mouth. Clin. Otolaryngol. 34:358–363. 19673984 10.1111/j.1749-4486.2009.01930.x

[22] Johnsson, M. , M. Winder , H. Zawia , I. Lodoen , G. Tobin , and B. Gotrick . 2016 In vivo studies of effects of antidepressants on parotid salivary secretion in the rat. Arch. Oral Biol. 67:54–60. 27023402 10.1016/j.archoralbio.2016.03.010

[23] Chen, J. , P. Liu , Q. Wang , L. Wu , and X. Zhang . 2015 Influence of intensity‐modulated radiation therapy on the life quality of patients with nasopharyngeal carcinoma. Cell Biochem. Biophys. 73:731–736. 27259317 10.1007/s12013-015-0638-0

[24] Visvanathan, V. , and P. Nix . 2010 Managing the patient presenting with xerostomia: a review. Int. J. Clin. Pract. 64:404–407. 19817913 10.1111/j.1742-1241.2009.02132.x

[25] Furness, S. , H. V. Worthington , G. Bryan , S. Birchenough , and R. McMillan . 2011 Interventions for the management of dry mouth: topical therapies. Cochrane Database Syst. Rev. 12:1–106. 10.1002/14651858.CD008934.pub2 PMC13266506 22161442

